# Lipoprotein lipase activity is required for cardiac lipid droplet production[S]

**DOI:** 10.1194/jlr.M043471

**Published:** 2014-04

**Authors:** Chad M. Trent, Shuiqing Yu, Yunying Hu, Nathan Skoller, Lesley A. Huggins, Shunichi Homma, Ira J. Goldberg

**Affiliations:** * Division of Preventive Medicine and Nutrition, Columbia University College of Physicians and Surgeons, New York, NY 10032; † Institute of Human Nutrition, Columbia University College of Physicians and Surgeons, New York, NY 10032; § Division of Cardiology, Columbia University College of Physicians and Surgeons, New York, NY 10032

**Keywords:** PPAR, Cd36, triglyceride

## Abstract

The rodent heart accumulates TGs and lipid droplets during fasting. The sources of heart lipids could be either FFAs liberated from adipose tissue or FAs from lipoprotein-associated TGs via the action of lipoprotein lipase (LpL). Because circulating levels of FFAs increase during fasting, it has been assumed that albumin transported FFAs are the source of lipids within heart lipid droplets. We studied mice with three genetic mutations: peroxisomal proliferator-activated receptor α deficiency, cluster of differentiation 36 (CD36) deficiency, and heart-specific LpL deletion. All three genetically altered groups of mice had defective accumulation of lipid droplet TGs. Moreover, hearts from mice treated with poloxamer 407, an inhibitor of lipoprotein TG lipolysis, also failed to accumulate TGs, despite increased uptake of FFAs. TG storage did not impair maximal cardiac function as measured by stress echocardiography. Thus, LpL hydrolysis of circulating lipoproteins is required for the accumulation of lipids in the heart of fasting mice.

The human heart will accumulate TGs in lipid droplets in disease states such as obesity and diabetes. Whether TG storage directly leads to reduced heart function, i.e., lipotoxicity (1, 2), or is a marker for accumulation of other toxic lipids is unclear. Evidence suggesting that stored TGs in cardiomyocytes are not always toxic has come from experiments in genetically modified mice. For instance, overexpression of the final enzyme in TG synthesis, diacylglycerol acyltransferase (DGAT)1, in cardiomyocytes increased TG stores but reduced accumulation of toxic lipids and did not reduce heart function (3). Overexpression of DGAT1 in skeletal muscle also increased TG storage in mice with diet-induced obesity and mimicked the “athlete's paradox” observed in endurance-trained humans; skeletal muscle DGAT1 transgenic mice had increased FA oxidation and improved insulin sensitivity (4). In contrast, increased TG accumulation in the human heart correlates with reduced heart function (5, 6). Moreover, greater TG stores are often associated with greater FA oxidation and greater injury during ischemia/reperfusion in isolated perfused hearts (7). Thus, the role of TG stores in the heart is unclear.

Even the substrate used for heart TG production has not been established. One physiologic stimulus that causes lipid accumulation in mouse hearts is prolonged fasting (8). Because starvation is a threat to survival, lipid accumulation in the heart may be an adaptation to accommodate future energetic demands, to protect the heart from lipotoxicity, or to do both. Understanding the features of this adaptation may provide insight into mechanisms that drive lipid accumulation under physiologic and pathological conditions. During fasting, animals rely exclusively on stored energy. While the liver produces and releases glucose under fasting conditions, this is insufficient to meet the energetic demands of the body (9). Adipose tissue is the major storage depot for energy in the form of TGs. During the fed state, dietary glucose stimulates insulin secretion, which simultaneously promotes glucose utilization and lipid storage. During fasting, circulating insulin levels fall while glucagon and catecholamines increase. This shift in the hormonal milieu leads to an activation of glycogenolysis in the skeletal muscle and liver and lipolysis in the adipose tissue. However, prolonged fasting will deplete glycogen stores and thus the energy demands of peripheral tissues must rely on both the liver, to secrete glucose and ketone bodies, and TGs and adipose tissue, to secrete FFAs and glycerol. Adipose tissue secreted glycerol, as well as lactate secreted from both adipose tissue and muscle, are taken up by the liver and used as substrates for gluconeogenesis.

We tested to determine whether reduced FA oxidation increased fasting-induced TG accumulation in the heart. To do this we studied PPARα knockout mice. Surprisingly, we found that fasted *Ppara*^−/−^ mice had no lipid droplet accumulation in hearts and had a marked reduction in mRNA levels of the FA transporter cluster of differentiation 36 (CD36) as well as lipoprotein lipase (LpL) (10, 11); LpL is required for heart uptake of FFAs from lipoprotein TGs. We then assessed the specific roles of CD36 and LpL in heart TG accumulation. Our data show that LpL activity is required for the accumulation of heart lipid droplets. In addition, we demonstrated that lipid droplet accumulation does not affect maximal systolic function of the heart.

## MATERIALS AND METHODS

### Animals and fasting

We used 3–4-month-old male C57BL/6 mice, *Ppara*^−/−^ mice (12), *Cd36*^−/−^ mice (13), floxed LpL mice (LpL^flox/flox^), and heart-specific LpL knockout (hLpL0) mice (14). Mice were raised on a normal chow diet. C57BL/6 mice were used as controls for both *Ppara*^−/−^ and *Cd36*^−/−^ mice and LpL^flox/flox^ littermates served as controls for the hLpL0 studies. Mice of each genotype were divided into two groups. One group was subjected to a 16 h overnight fast and the other group was fed ad libitum over the same time period. These mice were then euthanized with a lethal injection of 100 mg/kg ketamine and 10 mg/kg xylazine. All procedures were approved by the Columbia University Institutional Animal Care and Use Committee.

### Tissue collection

A ventral incision was made after administration of ketamine-xylazine. The left ventricle of the heart was perfused with 10 ml of PBS or until the liver appeared blanched. Tissues were rapidly removed and frozen in liquid nitrogen. Heart pieces were embedded into Tissue-Tek OCT compound (Sakura) for histology.

### Measurement of plasma lipids and glucose

Two hundred microliters of blood were drawn from each animal and then centrifuged at 2,000 rpm for 10 min to obtain plasma. Plasma was utilized for measurement of TGs, FFAs, and glucose by colorimetric assays. TG measurements were made using the Thermo Scientific Infinity assay (Thermo Scientific), FFAs were measured using the Wako NEFA kit, and plasma glucose was measured using the Wako Autokit Glucose kit (Wako Life Sciences).

### Lipid extraction from tissues

The lipid extraction protocol was adapted from the Folch method (15). Approximately 100 mg of tissue in 1 ml of PBS were homogenized using stainless steel beads for 1 min in a bead beater homogenizer. From each sample, 50 μl were removed for protein analysis and 3 ml of 2:1 chloroform:methanol was added to the rest and vortexed. Samples were then centrifuged for 10 min at 3,000 rpm at 4°C. The lower organic phase was then collected and dried under nitrogen gas. The dried lipid was then dissolved in 500 μl of 1% Triton X-100 in chloroform, further dried, and then dissolved in 100 μl of double distilled water.

### Lipid and protein measurements of tissues

The sample of tissue lysate retained from the lipid extraction protocol was assayed for protein content using Bradford reagent (Bio-Rad) following the instructions of the manufacturer. Using the tissue lipid extract, assays for TGs and FFAs were performed as previously described for plasma lipids. Lipid measurements were normalized to protein content or tissue weight.

### Microscopy for cardiac lipid visualization

Heart pieces were embedded in Tissue-Tek OCT compound (Sakura) and then air dried and fixed with formalin. Sections were washed with distilled water and isopropanol. Lipids were then stained with Oil Red O for 18 min, washed with isopropanol and distilled water, and then counterstained with hematoxylin. Slides were once again washed with distilled water and covered with clear nail polish. Images were taken using a Leica DMLB microscope and digital camera. Representative images obtained from five animals of each genotype are shown.

### Glycogen staining and quantification

Periodic acid-Schiff (PAS) reagent staining was used to demonstrate heart glycogen. Sections of OCT embedded hearts were placed in 10% neutral buffered formalin. Ventricular tissue sections were fixed in methanol for 10 min and stained with PAS reagent (Poly Scientific), hematoxylin, and eosin. Images were taken using a Leica DMLB microscope and digital camera. Four to five mouse hearts were used for each genotype and for each feeding condition, and several representative images were captured for each mouse.

Glycogen was also measured by extracting total insoluble carbohydrates and digesting with amyloglucosidase; free glucose was then measured and reported as ratio to tissue weight used for measurement as previously described (16). Ventricular tissue was hydrolyzed in 300 μl of 5.4 M KOH in a 100°C water bath for 30 min. Then, 100 μl 1 M Na_2_SO_4_ and 800 μl of 100% ethanol were added to each sample. Samples were boiled for 5 min and then centrifuged at 10,000 *g* for 5 min. The glycogen pellet was dissolved in 200 μl water and ethanol precipitation was performed twice with addition of 800 μl of 100% ethanol. Finally, the glycogen pellet was dissolved in 200 μl of 60 U/ml amyloglucosidase (Sigma) in 0.2 M sodium acetate (pH 4.8) and incubated for 3 h at 40°C. Each sample was diluted five times and glucose concentration was measured using the Wako Autokit Glucose kit (Wako Life Sciences).

### Cardiac gene expression

Total RNA was purified from a 30–50 mg piece of heart using TRIzol reagent (Invitrogen) according to the instructions of the manufacturer. cDNA was synthesized using the SuperScript III First-Strand Synthesis SuperMix (Invitrogen) and quantitative real-time PCR was performed with SYBR Green PCR Core reagents (Agilent Technologies) using an Mx3000 sequence detection system (Stratagene, La Jolla, CA). Genes of interest were normalized against 18s rRNA. Primer sequences are listed in supplementary Table I.

### Western blotting

Hearts were excised as previously described. Approximately 20 mg of tissue was homogenized in RIPA buffer containing protease inhibitor cocktail (Sigma-Aldrich). Twenty-five micrograms of protein extract was applied to SDS-PAGE and transferred onto polyvinylidene fluoride membranes. Antibodies for perilipin (PLIN)2 and PLIN5 were obtained from Santa Cruz Biotechnology (PLIN2, β-actin) and American Research Products (PLIN5). Band density measurements were made using ImageJ software. PLIN2 and PLIN5 band densities were normalized to β-actin band density.

### Stress echocardiography

Echocardiography was performed on 3–4-month-old male *Cd36*^+/+^ (wild-type), *Cd36*^−/−^, LpL^flox/flox^, and hLpL0 mice fasted for 16 h. Two-dimensional echocardiography was performed using a high-resolution imaging system with a 30 MHz imaging transducer (Vevo 770; VisualSonics) in unconscious mice. The mice were anesthetized with 1.5–2% isoflurane and thereafter maintained on 0.5% isoflurane throughout the procedure. Care was taken to minimize sedation by monitoring the heart rate of the mice. Two-dimensional echocardiographic images were obtained using short-axis views at the level of papillary muscles, and each parameter was measured using M-mode view. Images were analyzed offline by a researcher blinded to the murine genotype. Left ventricular end-diastolic dimension (LVEDd) and left ventricular end-systolic dimension (LVEDs) were measured. Percentage fractional shortening (FS), which quantifies contraction of the ventricular wall and is an indication of muscle function, was calculated as FS = ([LVEDd − LVEDs]/LVEDd) × 100. To assess stress response, 0.3 mg/kg isoproterenol (Sigma-Aldrich) was administered intraperitoneally. Successful administration of drug was confirmed by observation of increased heart rate.

### Pharmacologic inhibition of LpL

Poloxamer 407 (P407) was prepared in PBS as previously described (17). Mice were injected intraperitoneally with 1 mg/g body weight of P407 and then fasted for 16 h. Control mice were injected with an equivalent volume of PBS. Mice were euthanized and analyzed as previously described.

### In vivo assessment of cardiac glucose and FFA uptake

FFA and glucose uptake were assessed in mice that were injected with PBS or P407 and then fasted for 16 h. [9,10-^3^H(N)]oleate (PerkinElmer Life Sciences) was complexed to 6% FA-free BSA (Sigma). Mice were injected intravenously with 1 μCi [9,10-^3^H(N)]oleate-BSA and blood was collected at 0.5, 1, 3, and 5 min after injection. Five minutes after injection, the body cavity was perfused with 10 ml of PBS by cardiac puncture and tissues were excised. Tissue was homogenized in PBS and radioactive counts were measured. Basal glucose uptake was measured in hearts following an intravenous administration of 2.5 μCi of 2-deoxy-D-[1-^14^C]glucose (PerkinElmer Life Sciences). Blood was collected 2, 30, and 60 min following injection. At 60 min, hearts were perfused with PBS, tissues were excised, and radioactive counts were measured. For all turnover studies, radioactivity per gram of tissue was normalized to the respective 30 s or 2 min plasma counts (injected dose).

### Statistical analysis

Data are expressed as mean ± SE. Data were analyzed by the use of unpaired Student's *t*-test or two-way ANOVA.

## RESULTS

### Fasted *Ppara*^−/−^ mice do not store lipids in the heart

**Fig. 1.**
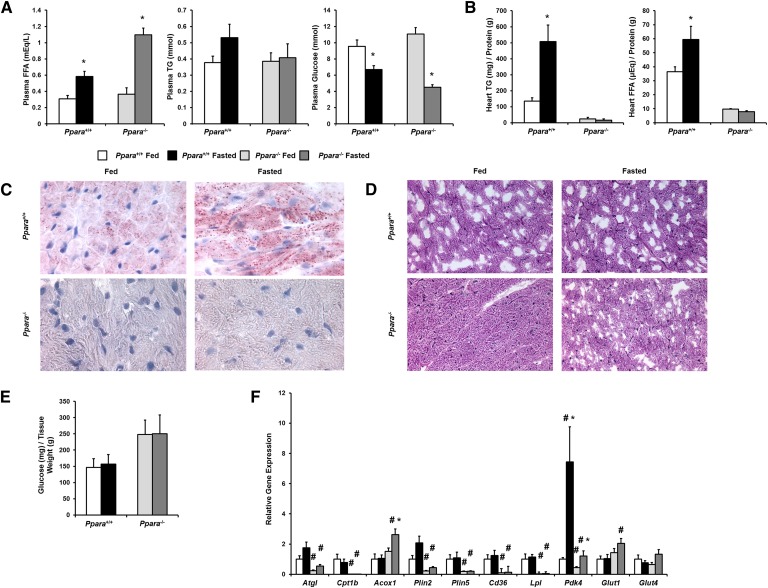
Overnight fasting of *Ppara*^+/+^ and *Ppara*^−/−^ mice. A: Plasma FFAs, TGs, and glucose were measured in 3–4-month-old male *Ppara*^+/+^ (n = 9) and *Ppara*^−/−^ (n = 5) mice that were fed or fasted for 16 h. Data were compared by Student's *t*-test. *P < 0.05. B: Total lipids were extracted from hearts, and TGs and FFAs were measured. Lipid content was normalized to protein content. Data were compared by Student's *t*-test. *P < 0.05. C: Heart sections of fed and fasted *Ppara*^+/+^ and *Ppara*^−/−^ mice (n = 5) were stained with Oil Red O, indicating neutral lipid content (1,000× magnification). D: Heart sections of fed and fasted *Ppara*^+/+^ and *Ppara*^−/−^ mice (n = 5) were stained with PAS reagent, indicating glycogen content (400× magnification). E: Total glycogen was extracted from hearts of fed and fasted *Ppara*^+/+^ and *Ppara*^−/−^ mice and digested with amyloglucosidase, and free glucose was measured. Glucose content was normalized to tissue weight. F: Gene expression of *Atgl*, *Cpt1b*, *Acox1*, *Plin2*, *Plin5*, *Cd36*, *Lpl*, *Pdk4*, *Glut1*, and *Glut4* was assessed using quantitative real-time PCR. Gene expression is expressed relative to fed *Ppara*^+/+^ mice. Data were compared by two-way ANOVA. *P < 0.05 compared within genotype; #*P* < 0.05 compared with *Ppara*^+/+^ mice of same feeding status.

We first assessed heart lipid storage in *Ppara*^−/−^ mice. We fasted *Ppara*^+/+^ (wild-type) and *Ppara*^−/−^ mice overnight for 16 h. Fasting increased plasma FFAs 2-fold in *Ppara*^+/+^ mice and 3-fold in *Ppara*^−/−^ mice, but had no significant effect on plasma TGs (**Fig. 1A**). Plasma glucose decreased approximately 30% in fasted *Ppara*^+/+^ mice and approximately 60% in fasted *Ppara*^−/−^ mice. Fasting increased heart TGs 5-fold in *Ppara*^+/+^ mice, but there was no significant TG accumulation in *Ppara*^−/−^ mice (Fig. 1B). Heart FA levels increased approximately 30% in *Ppara*^+/+^ mice, but were not increased in *Ppara*^−/−^ mice (Fig. 1B). Fasted *Ppara*^+/+^ mice had increased Oil Red O staining, but *Ppara*^−/−^ mice had minimal staining (Fig. 1C).

We then assessed heart glycogen storage in *Ppara*^−/−^ mice to determine whether these hearts depleted their stored carbohydrate. There was no difference in PAS reagent staining of glycogen (Fig. 1D) or extracted glycogen content (Fig. 1E) after fasting. There tended to be increased glycogen in *Ppara*^−/−^ hearts both before and after fasting.

Changes in genes required for lipid and glucose metabolism were determined in hearts of fed and fasted mice. Adipose TG lipase (*Atgl*), the rate limiting enzyme for intracellular TG lipolysis (18), was decreased by 50% in both fed and fasted *Ppara*^−/−^ mice compared with *Ppara*^+/+^ mice (Fig. 1F). Expression of carnitine palmitoyl transferase (*Cpt*)*1b*, the rate limiting enzyme for mitochondrial lipid oxidation, was minimal in both fed and fasted *Ppara*^−/−^ hearts. Surprisingly, mRNA of acyl CoA oxidase (*Acox*)*1*, the first enzyme in the lipid oxidation pathway, was increased in fasted *Ppara*^−/−^ hearts. However, decreased FA oxidation has been previously observed in these hearts (19, 20).

Therefore, absence of TG stores was not likely due to increased FA oxidation. As expected, mRNA levels of lipid droplet protein genes *Plin2* and *Plin5* were minimal in both fed and fasted *Ppara*^−/−^ mice compared with *Ppara*^+/+^ mice (21).

Fasting dramatically increased expression of pyruvate dehydrogenase kinase (*Pdk*)*4*, the negative regulator of glucose oxidation, in hearts from *Ppara*^+/+^ mice. Fasted *Ppara*^−/−^ mice also had increased *Pdk4* mRNA levels compared with fed *Ppara*^−/−^ mice, but these levels were still reduced compared with the *Ppara*^+/+^ counterparts. Fasted *Ppara*^−/−^ mice had increased mRNA expression of glucose transporter (*Glut*)*1*, the insulin insensitive glucose transporter, compared with fasted *Ppara*^+/+^ hearts, but there was no difference in expression of *Glut4*, the insulin sensitive glucose transporter. Most remarkable was that fed and fasted *Ppara*^−/−^ hearts had an ∼80% reduction in lipid uptake genes *Cd36* and *Lpl* (Fig. 1F).

We assessed heart mRNA levels of several genes involved in both de novo lipogenesis and TG formation. Expression of acetyl-CoA carboxylase (*Acc*)*2*, which is rate-limiting for de novo lipogenesis, was increased in the hearts of fed *Ppara*^−/−^ mice (supplementary Fig. IIIA). mRNA expression of *Fasn*, the second rate-limiting enzyme for de novo lipogenesis, was increased in the hearts of both fed and fasted *Ppara*^−/−^ mice. mRNA levels of *Dgat1*, the rate limiting enzyme for TG synthesis, were decreased in hearts from both fed and fasted *Ppara*^−/−^ mice. We also measured gene expression of FA transporters other than *Cd36*. Hearts from both fed and fasted *Ppara*^−/−^ mice had decreased expression of FA transport protein 1 (*Slc27a1*, or *Fatp1*), but increased expression of FA binding protein-plasma membrane (*Got2*, or *FABPpm*). Finally, we measured TG lipase activity in hearts of fed and fasted *Ppara*^+/+^ and *Ppara*^−/−^ mice. Both fed and fasted *Ppara*^−/−^ mice had 80–90% increased heart TG lipase activity compared with *Ppara*^+/+^ mice of the same nutritional status (supplementary Fig. IVA).

### Fasted *Cd36*^−/−^ mice do not store lipids in the heart

**Fig. 2.**
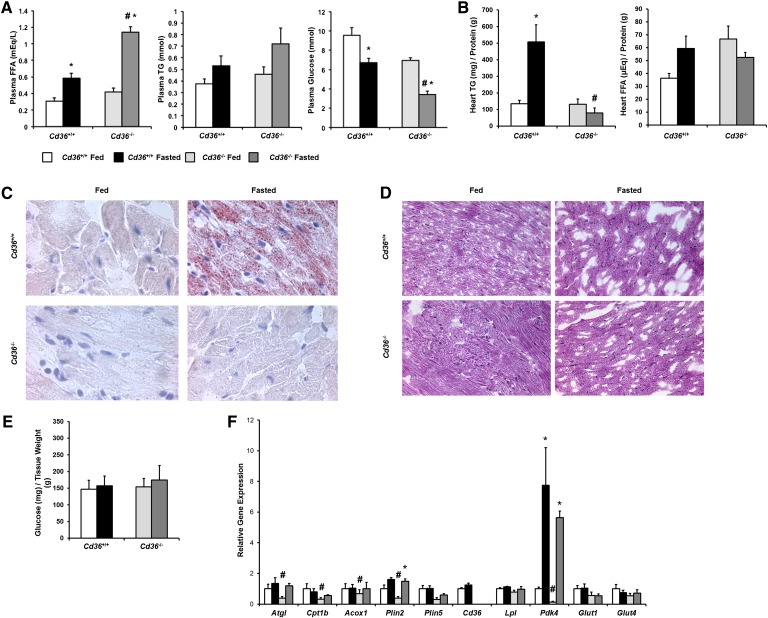
Overnight fasting of *Cd36*^+/+^ and *Cd36*^−/−^ mice. A: Plasma FFAs, TGs, and glucose were measured in 3–4-month-old male *Cd36*^+/+^ (n = 9) and *Cd36*^−/−^ (n = 9) mice that were fed or fasted for 16 h. Data were compared by 2-way ANOVA. *P < 0.05 compared within genotype, #*P* < 0.05 compared to *Cd36*^+/+^ mice of same feeding status. B: Total lipids were extracted from hearts of *Cd36*^+/+^ and *Cd36*^−/−^ mice, and TGs and FFAs were measured. Lipid content was normalized to protein content. Data were compared by 2-way ANOVA. *P < 0.05 compared within genotype, #*P* < 0.05 compared to *Cd36*^+/+^ mice of same feeding status. C: Heart sections of fed and fasted *Cd36*^+/+^ and *Cd36*^−/−^ mice (n = 5) were stained with Oil Red O (1,000× magnification). D: Heart sections of fed and fasted *Cd36*^+/+^ and *Cd36*^−/−^ mice (n = 5) were stained with PAS reagent (400× magnification). E: Total glycogen was extracted from hearts of fed and fasted *Cd36*^+/+^ and *Cd36*^−/−^ mice and digested with amyloglucosidase, and free glucose was measured. Glucose content was normalized to tissue weight. F: Gene expression of *Atgl*, *Cpt1b*, *Acox1*, *Plin2*, *Plin5*, *Cd36*, *Lpl*, *Pdk4*, *Glut1*, and *Glut4* was assessed using quantitative real-time PCR. Gene expression is expressed relative to fed *Cd36*^+/+^ mice. Data were compared by two-way ANOVA. *P < 0.05 compared within genotype; #*P* < 0.05 compared with *Cd36*^+/+^ mice of same feeding status.

Next, we determined whether CD36 deficiency would be sufficient to prevent heart TG accumulation during the fasted state. Fasting increased plasma FFAs 3-fold in *Cd36*^−/−^ mice (**Fig. 2A**). Plasma TGs tended to be higher in fasted *Cd36*^−/−^ mice compared with *Cd36*^+/+^ mice. Fasted *Cd36*^−/−^ mice had an ∼50% decrease in plasma glucose. There was no significant TG accumulation in *Cd36*^−/−^ hearts (Fig. 2B, C). Heart FA content also did not increase in hearts from *Cd36***^−/−^** mice (Fig. 2B). Glycogen content was similar in all hearts (Fig. 2D, E). Finally, *Cd36*^−/−^ mouse hearts tended to have decreased expression of lipid metabolism genes (*Atgl*, *Cpt1b*, *Acox1*, *Atgl*, *Plin2*, and *Plin5*) in the fed state, but fasted *Cd36*^−/−^ mouse hearts had similar gene expression to hearts of *Cd36*^+/+^ mice (Fig. 2F). *LpL* mRNA levels were comparable to control mice in both fasting and fed hearts. Glucose oxidation and uptake genes (*Pdk4*, *Glut1*, and *Glut4*) were comparable between genotypes and feeding conditions. Fed and fasted *Cd36*^−/−^ mice had decreased expression of *Dgat2* (supplementary Fig. IIIB). Hearts of fasted *Cd36*^−/−^ mice had increased expression of *Slc27a1*, and both fed and fasted hearts had decreased expression of FA binding protein (*Fabp*)*3*. There were no differences in TG lipase activity between feeding conditions or genotype (supplementary Fig. IVB).

### Fasted hLpL0 mice do not store lipids in the heart

**Fig. 3.**
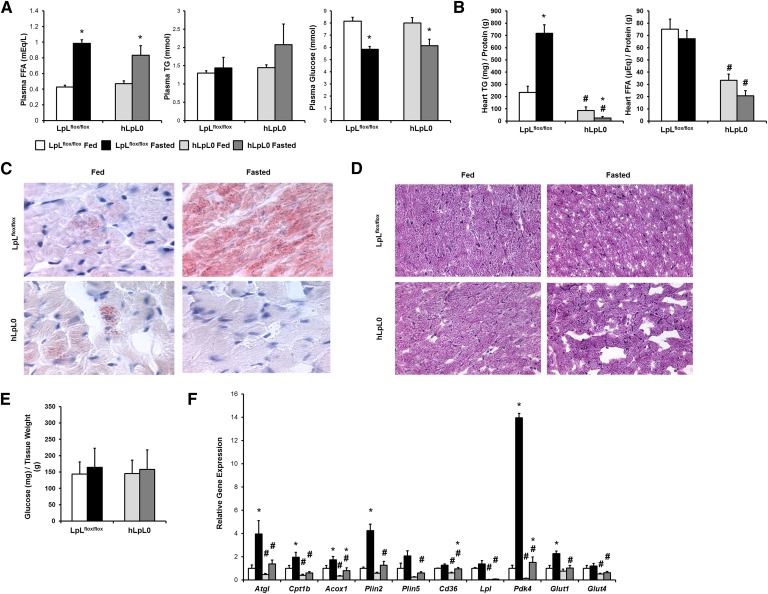
Overnight fasting of LpL^flox/flox^ and hLpL0 mice. A: Plasma FFAs, TGs, and glucose were measured in 3–4-month-old male LpL^flox/flox^ (n = 5) and hLpL0 (n = 4) mice that were fed or fasted for 16 h. Data were compared by 2-way ANOVA. *P < 0.05 compared within genotype, #*P* < 0.05 compared with LpL^flox/flox^ mice of same feeding status. B: Total lipids were extracted from hearts of LpL^flox/flox^ and hLpL0 mice, and TGs and FFAs were measured. Lipid content was normalized to protein content. Data were compared by 2-way ANOVA. *P < 0.05 compared within genotype, #*P* < 0.05 compared with LpL^flox/flox^ mice of same feeding status. C: Heart sections of fed and fasted LpL^flox/flox^ and hLpL0 mice (n = 5) were stained with Oil Red O (1,000× magnification). D: Heart sections of fed and fasted LpL^flox/flox^ and hLpL0 mice (n = 5) were stained with PAS reagent (400× magnification). E: Total glycogen was extracted from hearts of fed and fasted LpL^flox/flox^ and hLpL0 mice and digested with amyloglucosidase, and free glucose was measured. Glucose content was normalized to tissue weight. F: Gene expression of *Atgl*, *Cpt1b*, *Acox1*, *Plin2*, *Plin5*, *Cd36*, *Lpl*, *Pdk4*, *Glut1*, and *Glut4* was assessed using quantitative real-time PCR. Gene expression is expressed relative to fed LpL^flox/flox^ mice. Data were compared by two-way ANOVA. *P < 0.05 compared within genotype; #*P* < 0.05 compared with LpL^flox/flox^ mice of same feeding status.

If circulating FFAs are the source of heart TG stores during fasting, we would expect that loss of lipoprotein TG hydrolysis in the heart would not affect lipid droplet accumulation during fasting. To test this, we fasted hLpL0 mice and compared them to LpL^flox/flox^ littermates. After fasting hLpL0 mice had normal increases in plasma FFA levels, an approximately 2-fold increase (**Fig. 3A**). hLpL0 mice tended to have slightly elevated TGs in the fasted state, as has been reported (22). Plasma glucose fell 20% in both LpL^flox/flox^ and hLpL0 mice. Surprisingly, hLpL0 mice did not accumulate cardiomyocyte TGs during fasting (Fig. 3B, C). Glycogen content was similar in all hearts (Fig. 3D, E).

We predicted that changes in gene expression in the fasting hLpL0 hearts would explain the lack of TG stores. mRNA levels of *Atgl*, *Cpt1b*, and *Acox1* were reduced in both fed and fasted hLpL0 mouse hearts (Fig. 3F), consistent with the reduction in FA oxidation in these hearts (23). hLpL0 mice also had decreased expression of *Plin2* and *Plin5* compared with fasted LpL^flox/flox^ mice. Although these hearts do not have reduced FFA uptake, *Cd36* mRNA was reduced in both fed and fasted hLpL0 hearts. *Pdk4* expression was reduced in both fed and fasted hLpL0 hearts compared with LpL^flox/flox^ hearts. Although glucose uptake was increased in hLpL0 hearts (23), fasted *Glut1* mRNA levels were lower than controls and did not differ between fed and fasted hLpL0 mouse hearts. *Glut4* was also reduced in both fed and fasted hLpL0 hearts.

Hearts from both fed and fasted hLpL0 mice had decreased *Dgat1* mRNA expression (supplementary Fig. IIIC). Fasted hLpL0 mice had less *Dgat2* mRNA expression compared with fasted LpL^flox/flox^ mice. mRNA levels of the rate-limiting enzyme for production of monounsaturated FAs, stearoyl-CoA desaturase (*Scd*)*1*, were decreased in hearts of fasted hLpL0 mice. Expression of *Slc27a1* was increased in fasted floxed LpL mouse hearts, but not as much in fasted hLpL0 mouse hearts. FA transport protein 6 (*Fatp6*), *Fabp3*, and *Got2* were all decreased in hearts of fasted hLpL0 mice compared with fasted floxed LpL mice. There were no differences in TG lipase activity between feeding conditions or genotype (supplementary Fig. IVC).

### *Plin2* and *Plin5* protein expression is not affected by cardiac lipid stores

**Fig. 4.**
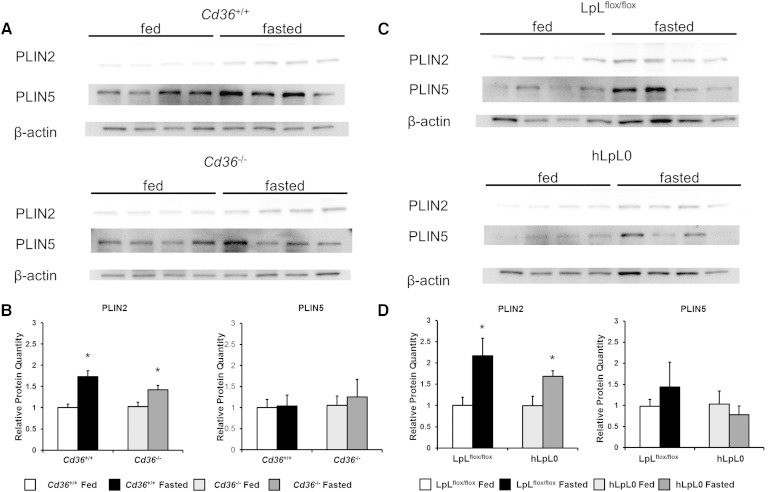
Western blot of PLIN2 and PLIN5 in hearts. A: PLIN2 and PLIN5 content was assessed with Western blotting in hearts of *Cd36*^+/+^ and *Cd36*^−/−^ mice. B: PLIN2 and PLIN5 protein content in hearts of *Cd36*^+/+^ and *Cd36*^−/−^ mice. Protein was quantified by band density measurements of the Western blot. Band densities were normalized to β-actin content within each sample. Data are expressed as relative amount compared with fed *Cd36*^+/+^ mice. Data were compared by Student's *t*-test. *P < 0.05. C: PLIN2 and PLIN5 content was assessed with Western blotting in hearts of LpL^flox/flox^ and hLpL0 mice. D: PLIN2 and PLIN5 protein content in hearts of LpL^flox/flox^ and hLpL0 mice was quantified by band density measurements of the Western blot. Data were normalized to β-actin content within each sample and expressed as relative amount compared with fed LpL^flox/flox^ mice. Data were compared by Student's *t*-test. *P < 0.05.

A reduction in PLIN2 prevents lipid accumulation in the liver (24, 25), and we noticed a reduction in *Plin2* mRNA. For this reason, we measured PLIN2 protein in the hearts of fed and fasted mice. Fasting increased PLIN2 protein in *Cd36*^+/+^ mice, and unexpectedly also in *Cd36*^−/−^ mouse hearts (**Fig. 4A**). Band density measurement indicated about a 40–50% increase in PLIN2 after fasting in both *Cd36*^+/+^ and *Cd36*^−/−^ mice (Fig. 4B). Because PLIN5 blocks TG lipolysis (26–28), a lack of change in mRNA but reduced protein could allow more rapid degradation of stored TGs. Therefore, we measured Plin5 protein in the fed and fasted state. PLIN5 protein levels were highly variable (Fig. 4A), but not significantly different between the fed and fasted states (Fig. 4B). Similar changes in both PLIN2 and PLIN5 protein were observed in hLpL0 mice (Fig. 4C, D).

### Blocking circulating TG degradation prevents cardiac lipid accumulation

**Fig. 5.**
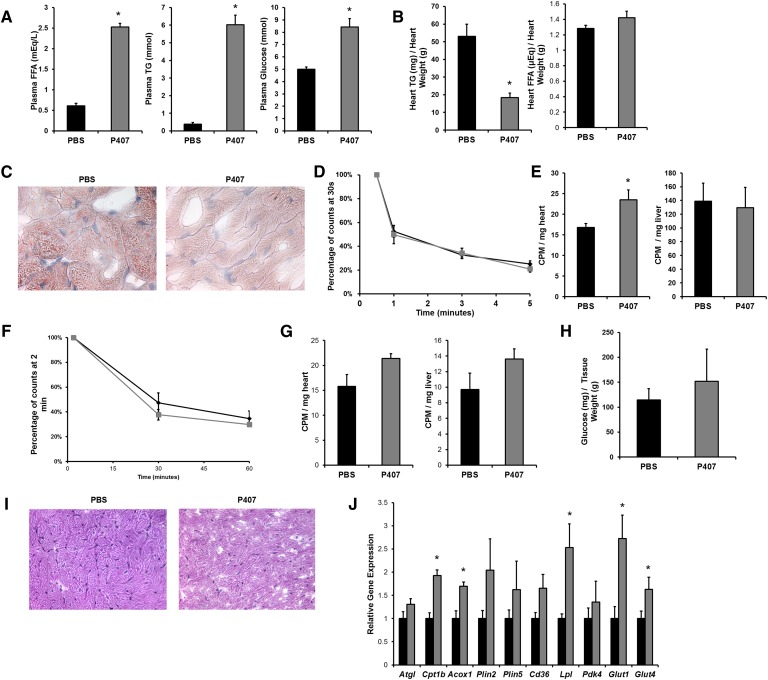
Overnight-fasted mice treated with P407. A: Plasma FFAs, TGs, and glucose in 3–4-month-old C57/BL6 male mice (n = 5) that were injected intraperitoneally with 1 mg/g P407 or an equivalent volume of PBS. Data were compared by Student's *t*-test. *P < 0.05. B: Total lipids were extracted from hearts of fasted PBS- and P407-treated mice, and TGs and FFAs were measured. Lipid content was normalized to heart weight. Data were compared by Student's *t*-test. *P < 0.05. C: Heart sections of fasted PBS- and P407-treated mice (n = 5) were stained with Oil Red O (1,000× magnification). D: Plasma disappearance of the [^3^H]oleate was measured at 30 s, 1 min, 3 min, and 5 min after injection. E: Cardiac and hepatic FFA uptake were assessed using [^3^H]oleate. Data were compared by Student's *t*-test. *P < 0.05. F: Plasma disappearance of the [^14^C]2-deoxyglucose was measured at 2 min, 30 min, and 60 min after injection. G: Cardiac and hepatic glucose uptake were assessed using [^14^C]2-deoxyglucose. H: Total glycogen was extracted from hearts of fasted PBS- and P407-treated mice and digested with amyloglucosidase, and free glucose was measured. Glucose content was normalized to tissue weight. I: Heart sections of fasted PBS- and P407-treated mice (n = 5) were stained with PAS reagent, indicating glycogen content (400× magnification). J: Gene expression of *Atgl*, *Cpt1b*, *Acox1*, *Plin2*, *Plin5*, *Cd36*, *Lpl*, *Pdk4*, *Glut1*, and *Glut4* was assessed using quantitative real-time PCR. Data were compared by Student's *t*-test. *P < 0.05.

Because of the surprising observation that hLpL0 mouse hearts did not accumulate TGs during fasting, a result suggesting that circulating TGs are the primary substrate for cardiac lipid accumulation, we used a drug that blocks lipolysis of circulating TGs. Mice were administered P407 and then fasted for 16 h. Plasma TGs increased 10-fold in the P407-treated mice, indicating a complete block of TG uptake (**Fig. 5A**). Plasma FFA and glucose levels were also higher in the P407-treated mice. Next, we looked at the heart lipids. As we found in the hLpL0 hearts, cardiac FFAs were not affected by P407 treatment, but P407-treated mice had 60% less TGs than PBS-treated mice (Fig. 5B). There was decreased Oil Red O staining of neutral lipids in P407-treated mouse hearts compared with PBS-treated mouse hearts (Fig. 5C).

We then measured uptake of circulating FFAs in control and P407-treated mice. Plasma turnover of the label was identical in the control and treated mice (Fig. 5D). Heart uptake of FFAs was greater in the lipolysis-inhibited mice (Fig. 5E), consistent with a greater reliance of the heart on FFAs than TGs. Liver uptake of the label did not differ between groups. Next, we measured uptake of circulating glucose in control and P407-treated mice. Plasma turnover of the labeled glucose in plasma was the same in both groups of mice (Fig. 5F). Uptake of plasma glucose tended to be increased in P407-treated mouse hearts (*P* = 0.08) and livers (Fig. 5G). Total glycogen content was not changed between groups (Fig. 5H, I).

Lipid metabolism genes tended to be increased with P407, but not all increases reached statistical significance. *Cpt1b*, *Acox1*, and *Lpl* were increased in P407-treated mice while increases in *Atgl*, *Plin2*, *Plin5*, and *Cd36* were less robust (Fig. 5J). *Pdk4* expression was not affected by P407-treatment, but *Glut1* and *Glut4* were both increased in hearts of P407-treated mice. P407-treated fasted mice had increased heart expression of *Fabp3* and tended to have increased expression of *Fatp6* (supplementary Fig. IIID). There was a 30% increase in TG lipase activity in fasted mice treated with P407 (supplementary Fig. IVD).

### Cardiac TG accumulation during fasting does not impair heart function

**Fig. 6.**
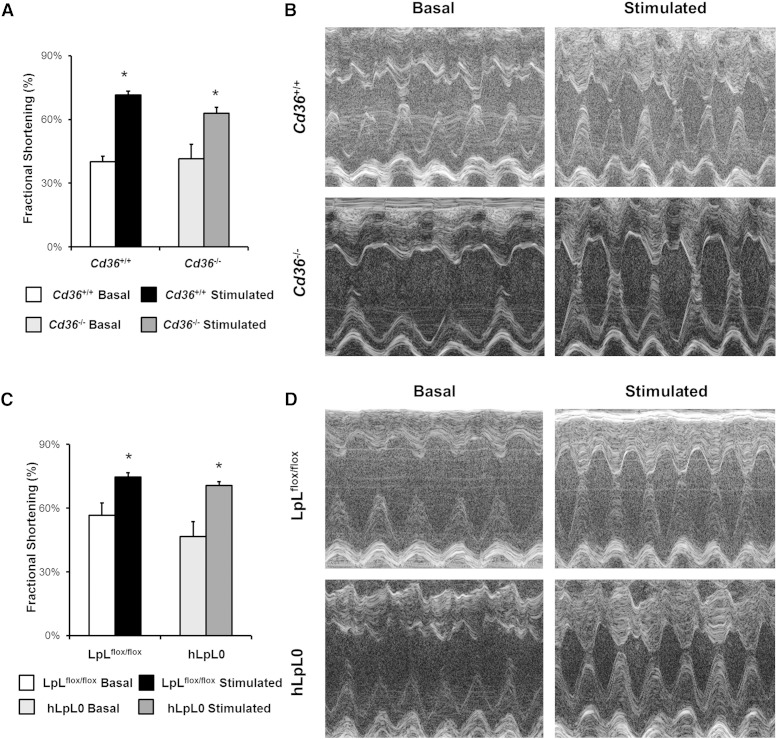
Stress echocardiography of fasted mice. A: Male *Cd36*^+/+^ (n = 10) and *Cd36*^−/−^ (n = 9) mice (3–4 months old) were fasted for 16 h and then injected intraperitoneally with 1.5 mg/kg isoproterenol. Cardiac function was assessed with M-mode echocardiography and FS was measured before and after injection of isoproterenol. Representative M-mode echocardiography images are pictured. Data were compared by Student's *t*-test. *P < 0.05. B: Male LpL^flox/flox^ (n = 7) and hLpL0 (n = 8) mice (3–4 months old) were fasted for 16 h and then injected intraperitoneally with 0.3 mg/kg isoproterenol. Cardiac function was assessed with M-mode echocardiography and FS was measured before and after injection of isoproterenol. Data were compared by Student's *t*-test. *P < 0.05.

Cardiac TG accumulation has been postulated to cause toxicity and to reduce heart function (2, 29). To determine whether TG accumulation during fasting affects cardiac function, we fasted mice overnight and measured FS before and after administration of isoproterenol. We used young mice, 3–4 months old, prior to the development of severe heart dysfunction that occurs in the hLpL0 mice (23). Basal FS was similar (∼40%) in fasted *Cd36^+/+^* and *Cd36*^−/−^ mice. Isoproterenol injection resulted in a maximum FS of 70% in fasted *Cd36*^+/+^ hearts and 60% in *Cd36*^−/−^ hearts (**Fig. 6A, B**). Basal FS tended to be greater in LpL^flox/flox^ mice (56%) compared with hLpL0 mice (47%), but LpL^flox/flox^ and hLpL0 mice had a similar maximal increase to 70% FS upon isoproterenol stimulation (Fig. 6C, D).

## DISCUSSION

In this series of experiments, we studied the source of FAs required for heart lipid droplet formation. We first found that hearts from *Ppara*^−/−^ mice did not accumulate lipid droplets, despite their increase in plasma FFAs and reduced FA oxidation. FFA uptake into the heart may be mediated by several transporters and nonreceptor-mediated movement of FFAs across the membrane (10, 11). The FA transporter CD36 facilitates a major fraction of the albumin-bound FFA uptake into cardiac cells (30), and also FFAs derived from LpL hydrolysis of VLDLs (31). Loss of either CD36 or LpL reduced lipid droplet stores. Because CD36 is downstream of LpL (31), we then used a chemical inhibitor of lipolysis to confirm that defective lipolysis in the presence of continued FFA uptake could prevent heart TG accumulation. In agreement with our conclusion, albumin-bound FFAs were shown to be more likely to be oxidized and chylomicron TG-derived FFAs more likely to be esterified (32). Our data contrast with studies in isolated hearts, in which supply of excess FFAs can drive lipid droplet formation (33, 34). However, in vivo the majority of circulating FAs are esterified as TGs and phospholipids (85–90%). Thus it would not be surprising that they also are a major supplier of heart FFAs for TG storage. Finally, we showed that TG accumulation during overnight fasting did not impair or improve stimulated cardiac function.

Lipid uptake, oxidation, and storage are controlled by the PPAR family of transcription factors. The family members, PPARα, PPARβ/δ, and PPARγ, have overlapping transcriptional control of lipid metabolism gene expression. Two mouse models of heart-specific PPAR overexpression, the alpha-myosin heavy chain (MHC)-PPARα and MHC-PPARγ transgenic mice, have increased lipid uptake, increased neutral lipid storage, and increased lipid oxidation associated with cardiac dysfunction (35, 36). Although *Ppara*^−/−^ mice have increased fasting levels of plasma FFAs (37), the accumulation of heart lipids during fasting had not been assessed. We hypothesized that *Ppara*^−/−^ mice, which have reduced FA oxidation in the heart (12), would accumulate more lipids during a prolonged fast. We found just the opposite; TG accumulation was drastically reduced. Was this due to a defect in lipid droplet production, greater lipolysis of the stored TGs, or reduced lipid uptake into these hearts? Because lipid uptake is upstream of lipid storage and turnover, it seems most likely that a deficiency of lipid uptake would precede any intracellular metabolic abnormalities.

There are several mouse models that do not accumulate cardiac lipids during fasting. Mice deficient in hormone-sensitive lipase (HSL), an intracellular TG and diacylglycerol lipase, do not accumulate cardiac lipids during fasting, presumably due to the reduced ability of the adipose tissue to lipolyze stored TGs and release FFAs (38). HSL knockout mice have lower fasting plasma FFA and TG levels and decreased heart FFA uptake (38). Conversely, cardiomyocyte-specific HSL-overexpressing mice also do not accumulate lipids after prolonged fasting because of rapid lipid droplet turnover (39). *Ppara*^−/−^ mice did not have an increase in ATGL expression; in fact, mRNA levels of ATGL were dramatically reduced.

Another modulator of intracellular TG lipolysis is the lipid droplet protein PLIN5. Recently, *Plin5*^−/−^ mice were described to have defective storage of lipids in the heart (28); PLIN5 is responsible for regulating ATGL activity, thus with PLIN5 deficiency intracellular lipase activity is constantly turned on. *Plin4* deletion resulted in a similar phenotype due to a consequent decrease in *Plin5* expression (40). Overexpression of PLIN5 increased heart TG content (26, 27). *Plin2*^−/−^ mice had decreased lipid accumulation in the liver, suggesting that Plin2 promotes lipid accumulation (24). As others have reported (21), we observed that *Plin2* and *Plin5* mRNA were dramatically reduced in *Ppara*^−/−^ mouse hearts. Therefore, defects in lipid droplet formation in *Ppara*^−/−^ mice could be due to lack of PLIN2 or PLIN5 or, more likely, the reductions in PLIN2 and PLIN5 are secondary to reduced PPARα activation. Surprisingly, intracellular TG lipase activities were dramatically increased in *Ppara*^−/−^ mice, perhaps due to decreased *Plin2* and *Plin5* expression. Others have demonstrated a fasting-induced increase of TG lipase activity in the hearts of wild-type animals (41); we did not find this to be the case.

mRNA levels for *Cd36* and *Lpl* were both markedly reduced in the *Ppara*^−/−^ mice. Therefore, we fasted mice that were deficient in CD36 or heart LpL. *Cd36*^−/−^ mice have decreased cardiac FFA uptake and decreased VLDL-TG uptake, whereas hLpL0 mice have normal to increased cardiac FFA uptake and decreased VLDL-TG uptake (31, 42). Because the concentration of circulating FFAs increases during fasting, and mice that do not mobilize adipose lipid stores during fasting do not accumulate cardiac lipids (38), we hypothesized that FFAs are the primary substrate for lipid accumulation in the hearts of fasted mice. We expected that *Cd36*^−/−^ mice would not form lipid droplets after an overnight fast, but hLpL0 mice would form lipid droplets. As expected, fasted *Cd36*^−/−^ mice failed to accumulate lipids in the heart. Surprisingly, the hLpL0 mice also failed to accumulate cardiac lipids.

We still believed that FFAs were driving this process, so we hypothesized that lipid droplets were not stabilized and were turned over more rapidly in hLpL0 mice. hLpL0 mice have decreased PPARα target gene expression (22, 43), so we suspected that lipid droplet proteins PLIN2 and PLIN5 were also reduced. We found that *Plin2* mRNA levels were reduced in both the fed and fasted state. However, *Plin5* mRNA was normal. As there is often a mismatch between *Plin2* and *Plin5* message and protein level, we assayed protein levels with Western blotting. Protein levels in the heart were comparable for each genotype, and fasting increased heart PLIN2 modestly in all genotypes. PLIN5 was comparable between control and hLpL0 mice with both feeding conditions. Finally, we measured heart intracellular TG lipase activity, and there was no difference between LpL^flox/flox^ mice and hLpL0 mice. Thus, we concluded that despite decreased PPARα target gene expression, it was unlikely that a defect in storage was preventing lipid accumulation in hLpL0 mice.

We then posited that VLDL-TGs, and not FFAs, were the source of the cardiac lipids for droplet accumulation observed in fasting. Indeed, cardiac LpL activity is increased after an overnight fast, suggesting an important role for circulating TGs in supplying the heart with lipids (44, 45). To test this hypothesis, we treated mice with P407, a detergent which interferes with lipolysis of circulating lipoproteins. P407 treatment dramatically reduced cardiac lipid accumulation, but did not reduce PPARα target gene expression. Furthermore, P407-treated mice had increased FFA uptake, yet still failed to form lipid droplets. However, the decrease in lipid droplet formation could be the result of increased intracellular TG lipase activity; it is unclear why intracellular TG lipase activity was increased in the P407-treated mice. We observed that P407 treatment and fasting together dramatically increased plasma FFAs; plasma FFA concentrations in P407-treated mice were 5-fold greater than those in plasma of nontreated fasting mice. This increase may be partially due to the partitioning of plasma FFAs onto VLDL particles (supplementary Fig. I). We concluded that TGs, and not FFAs, are the primary substrate for cardiac lipid accumulation observed in fasting, and that PPARα is necessary, but not sufficient, for cardiac lipid accumulation.

In these studies, we focused on lipid uptake pathways that modulate TG accumulation in the hearts of fasted animals. As the heart is a dynamic organ that possesses the ability to use multiple substrates, we asked whether hearts in fasted animals that have defective lipid uptake may have changes in glucose utilization. Mice that have decreased lipid uptake in cardiomyocytes tend to increase glucose uptake and catabolism (23, 46). We suspected that this might be happening in mice that failed to store lipid droplets in the cardiomyocytes. Heart gene expression of *Pdk4*, a negative regulator of glucose oxidation, was increased in all fasted mice, but to a lesser degree in mice that had deficiencies in lipid uptake. This likely indicates an increased reliance on glucose oxidation, i.e., pyruvate conversion to acetyl-CoA. Although gene expression of the glucose transporters GLUT1 and GLUT4 was variable, it has been previously demonstrated that *Ppara*^−/−^ mice (47), *Cd36*^−/−^ mice (48), and hLpL0 mice (23) all have increased cardiac glucose uptake. P407-treated fasting mice, which did not accumulate TGs in cardiomyocytes, tended to have increased glucose uptake. However, glycogen content of hearts was similar for all genotypes, regardless of feeding status. We should note that others have reported increased heart glycogen with fasting (49).

We would have liked to assess where the albumin-bound FFAs from adipose lipolysis and the FFAs from LpL's action on circulating TGs were going once they entered the cardiomyocyte. Different pools of circulating lipids, either FFAs or TGs, may enter the cardiomyocyte and be immediately oxidized, or they may be esterified as TGs and later oxidized, or some combination of the two. FA turnover in the heart is very rapid with hydrolysis of much the pool of FAs within 10 min (23). Thus, assessing the residual FAs from labeled FFAs or VLDL-TGs is challenging. There are a number of elegant studies that have used isolated perfused hearts to track uptake, oxidation, and esterification of labeled TGs in chylomicrons and VLDLs (29, 50–52). However, methods to study tracer uptake and oxidation in vivo and not in perfused hearts are not available. It is likely that LpL-mediated accumulation of heart TGs during fasting is reflective of the much greater amount of circulating lipids that reside in TG particles. The total amount of albumin-bound FFAs might be insufficient to alone promote heart lipid accumulation during fasting. In support of the importance of TGs as a source of heart lipids, lipotoxic mice that have excessive cardiac lipid accumulation are cured with cardiac-specific LpL knockout (43). Furthermore, it might also be that FAs from different sources diverge in their intracellular fate. In support of this, one study of isolated perfused hearts found that FFAs were primarily oxidized, while FAs from chylomicrons divided equally into oxidation and cellular storage (32).

We assessed intracellular TG lipase activity to determine whether increased turnover of lipid droplets might explain why we did not see fasting-induced cardiac lipid accumulation in *Ppara*^−/−^, *Cd36*^−/−^, hLpL0, and P407-treated mice. Surprisingly, we found there was dramatically increased TG lipase activity in *Ppara*^−/−^ mice, despite the reduction in *Atgl* mRNA. The deficiency of *Plin2* and *Plin5* that we and others have observed (21) in these mice could also indicate greater TG turnover. We also observed an increase in heart TG lipase activity in fasted mice treated with P407. However, there were no differences in heart TG lipase activity between *Cd36*^−/−^ or hLpL0 mice and wild-type or floxed controls.

Although our data and others implicate LpL and CD36 as the primary players in cardiac lipid accumulation, it is possible that mice deficient in these proteins might have a reduction in other lipid transporters or de novo lipogenesis. We assessed mRNA expression of a number of other genes and found that some of these genes had increased expression, but this was not sufficient to restore TG lipid droplet accumulation.

**Fig. 7.**
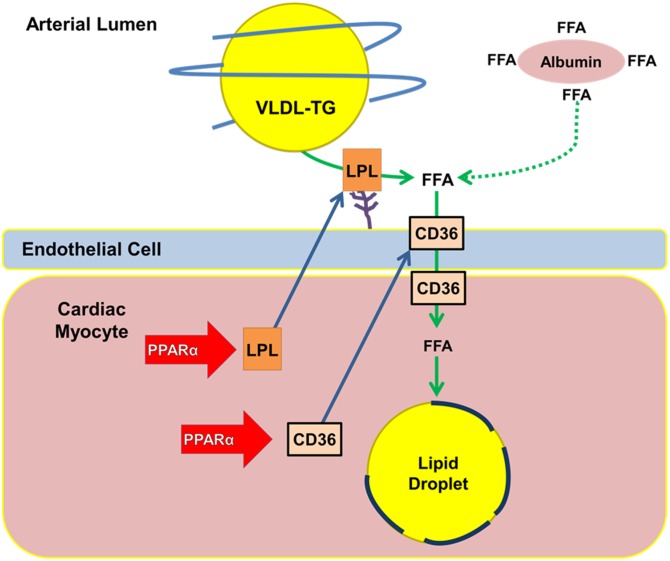
Pathways of lipid uptake leading to lipid droplet formation. PPARα drives lipid accumulation by regulating transcription of lipid uptake proteins LpL and CD36. Although both LpL and CD36 are required for cardiac lipid accumulation, LpL-mediated lipolysis of lipoprotein TGs is required for lipid droplet accumulation.

Lipid droplet accumulation is thought to occur due to an imbalance between lipid uptake and oxidation. This involves PPARα driven expression of LpL and CD36, allowing increased uptake of circulating lipoprotein TGs (**Fig. 7**). Whether TG accumulation is toxic is unresolved (3). Lipid droplets are found in hearts of patients with diabetes and metabolic syndrome (5, 6, 53) and in hearts of high-fat diet-fed rodents and genetically altered mice (2). Lipotoxicity can occur when ceramides, diacylglycerol, or other lipid species can alter cardiac cell signaling, disrupt membrane function, or cause apoptosis (54). We measured ceramides in hearts of fasted wild-type mice and did not observe an increase in ceramides coinciding with increased TG content (supplementary Fig. II).

Excessive FFA oxidation has been proposed to lead to mitochondrial dysfunction, apoptosis, and heart failure (7). For this reason, we tested to determine whether increased stored lipid would reduce stimulated heart function. It did not. We found that short-term starvation and consequent TG accumulation did not decrease cardiac output.

Although several models of lipid-induced heart dysfunction have massively increased amounts of heart TGs, others have increases similar to those we found with fasting. ATGL knockout mice have 20 times the amount of cardiac lipids at 12 weeks of age and dramatically decreased heart function leading to 50% mortality by 18 weeks of age (18). However, two groups recently reported that PLIN5 overexpression and a 3- to 10-fold increase in cardiac TGs led to only mild heart dysfunction (26, 27). Less dramatic increases in TG levels are occasionally associated with heart dysfunction, however, in those hearts diacylgycerols and ceramides are also increased (55). We have previously reported a model of 50% increased cardiac TGs in the fed state that is not associated with decreased cardiac function, the MHC-DGAT1 mouse model (3). In fact, this transgene reduces toxicity in other models without changing TG levels, but reducing heart ceramide 20% (3). In another model, transgenic MHC-PPARγ mice, a similar 2- to 3-fold increase in myocardial TGs but coupled to increased diacylglyceride and ceramide was associated with more than a 50% reduction in FS (8, 35, 56). The effects of diets and diabetes on heart TGs and other lipids and cardiac function are less clear. Three weeks of high-fat diet feeding increased heart TGs by 2- to 3-fold, but a 20% decrease of FS was only observed after 20 weeks of high-fat diet feeding (57, 58). Streptozotocin-diabetic mice had a 50% increase in cardiac TGs after 12 weeks associated with a 20% reduction of FS (59). *Ob*/*ob* and *db*/*db* mice all have varying degrees of increased cardiac TGs (from 2-fold to 4-fold) associated with heart dysfunction depending on the duration of the study (60, 61). Levels of ceramides and diacylglycerides were often not measured in these models. Increased heart TG content is sometimes, but not always, a hallmark of increased accumulation of other lipids, many of which are harmful to the heart.

Storage of TGs in the heart may be an adaptive response to decreased energy intake during starvation. However, it also occurs in the setting of pathological conditions including obesity, diabetes, and nonischemic heart failure (62, 63). Moreover, one report has suggested that heart lipid droplet accumulation occurs postischemia and is associated with more tissue damage (64). As we noted above, lipid droplet accumulation is sometimes associated with heart dysfunction and also with increased concentrations of potentially toxic lipids such as ceramides and diacylglycerols, but in other situations TG storage alone does not lead to heart dysfunction (2). Whether the association between heart TGs and function in humans is due to TGs or other accumulated lipids is not obvious. It should be noted that severe heart failure in humans leads to reduced heart TGs, but an increase in potentially toxic ceramides and diacylglycerols (65). Our data show that lipid droplets, at least those that occur during fasting, do not affect heart function.

In summary, our studies show that LpL lipolysis of TGs is required to store lipids in the hearts of fasting mice. This study further confirms previous work showing that hearts are active organs in TG metabolism. We now show that the very high level of cardiac LpL expression is involved not only in acquisition of circulating FAs for energy, but also for allowing storage.

## Supplementary Material

Supplemental Data
