# Supplementary material for: Lipoprotein lipase activity is required for cardiac lipid droplet production
Source: J Lipid Res. 2014 Apr;55(4):645–58. doi: 10.1194/jlr.M043471 (PMC3966699; doi:10.1194/jlr.M043471)
Supplement: Supplemental Data [file supp_55_4_645__index.html]

Lipoprotein Lipase Activity is Required for Cardiac Lipid Droplet Production — Lipoprotein lipase activity is required for cardiac lipid droplet production — Supplemental Data 

# Lipoprotein lipase activity is required for cardiac lipid droplet production

## Supplemental Data

**Files in this Data Supplement:**

- Supplemental figures - PDF of supplemental figures and tables
