# Supplementary material for: Lipoprotein lipase activity is required for cardiac lipid droplet production
Source: J Lipid Res. 2014 Apr;55(4):645–58. doi: 10.1194/jlr.M043471 (PMC3966699; doi:10.1194/jlr.M043471)
Supplement: Supplemental Data [file supp_M043471_jlr.M043471-1.pdf]

Lipoprotein Lipase Activity is Required for Cardiac Lipid Droplet Production

Chad M. Trent[1,2]; Shuiqing Yu[1]; Yunying Hu[1]; Nathan Skoller[2]; Lesley A. Huggins[1]; Shunichi Homma[3]; Ira J. Goldberg[1]

Supplemental Data

Supplemental figure 1. Distribution of plasma FFA in lipoprotein fractions

Plasma from PBS and P407-treated 16-hour fasted mice (n=4-5) was fractionated with density ultracentrifugation. Counts of [^{3}H]-oleate were measured in each lipoprotein fraction and reported as percentage of total counts in plasma. * indicates $p < 0.05$. Data were compared by Student's t-test.

Supplemental figure 2. Ceramide measurement in fed and fasted wild-type mice

Individual ceramide species and total ceramides were measured from hearts of fed and 16-hour fasted wild-type mice (n=9). * indicates $p < 0.05$ compared to fed mice. Data were compared by Student's t-test.

Supplemental figure 3. Lipid uptake, synthesis, and storage gene expression

A. Gene expression of *Slc27a1*, *Fatp6*, *Fabp3*, *Got2*, *Acc2*, *Fasn*, *Scd1*, *Dgat1*, *Dgat2*, and *Abhd5* was assessed using quantitative real-time PCR. Gene expression is expressed relative to fed *Ppara*$^{+/+}$ mice. * indicates $p < 0.05$ compared within genotype, # indicates $p < 0.05$ compared to *Ppara*$^{+/+}$ mice of same feeding status; data were compared by 2-way ANOVA. B. Gene expression of *Slc27a1*, *Fatp6*, *Fabp3*, *Got2*, *Acc2*,

Fasn, *Scd1*, *Dgat1*, *Dgat2*, and *Abhd5* was assessed using quantitative real-time PCR. Gene expression is expressed relative to fed *Cd36*$^{+/+}$ mice. * indicates $p < 0.05$ compared within genotype, # indicates $p < 0.05$ compared to *Cd36*$^{+/+}$ mice of same feeding status; data were compared by 2-way ANOVA. C. Gene expression of *Slc27a1*, *Fatp6*, *Fabp3*, *Got2*, *Acc2*, *Fasn*, *Scd1*, *Dgat1*, *Dgat2*, and *Abhd5* was assessed using quantitative real-time PCR. Gene expression is expressed relative to fed LpL$^{flox/flox}$ mice. * indicates $p < 0.05$ compared within genotype, # indicates $p < 0.05$ compared to LpL$^{flox/flox}$ mice of same feeding status; data were compared by 2-way ANOVA. D. Gene expression of *Slc27a1*, *Fatp6*, *Fabp3*, *Got2*, *Acc2*, *Fasn*, *Scd1*, *Dgat1*, *Dgat2*, and *Abhd5* was assessed using quantitative real-time PCR. * indicates $p < 0.05$, data were compared by Student's t-test.

Supplemental figure 4.

A. Heart tissue lysates from fed and fasted *Ppara*$^{+/+}$ and *Ppara*$^{-/-}$ mice (n=5) were incubated with ^{3}H-labelled triolein micelles to assess TG lipase activity. # indicates $p < 0.05$ compared to *Ppara*$^{+/+}$ mice of same feeding status; data were compared by 2-way ANOVA. B. Heart TG lipase activity was measured in fed and fasted *Cd36*$^{+/+}$ and *Cd36*$^{-/-}$ mice (n=5). C. Heart TG lipase activity was measured in fed and fasted LpL$^{flox/flox}$ and hLpL0 mice (n=5) D. Heart TG lipase activity was measured in fasted mice treated with either PBS or P407 (n=4-5). * indicates $p < 0.05$, data were compared by Student's t-test.

Supplemental Table 1

The sequences of mouse primers used for qRT-PCR gene expression analysis are reported.

Supplemental Figure 1

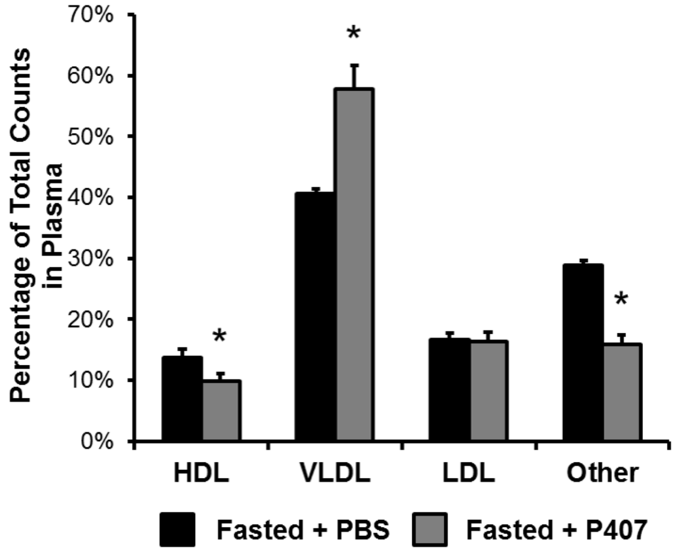

Supplemental Figure 2

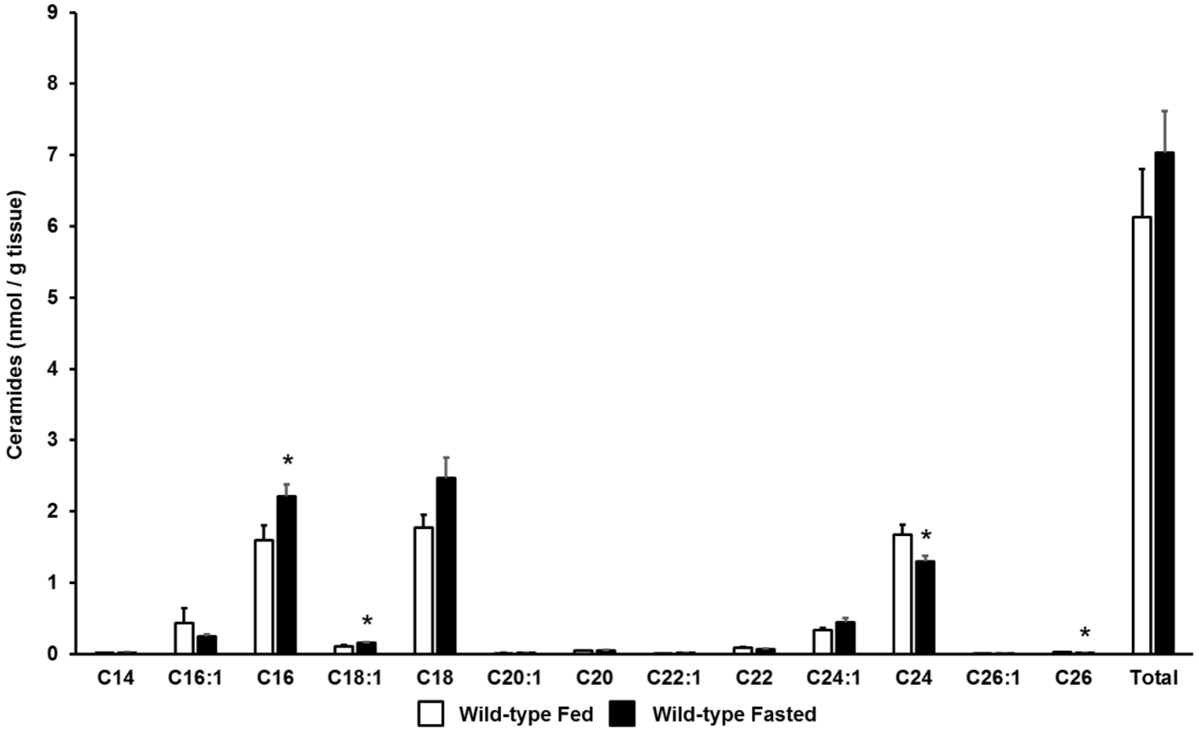

Supplemental Figure 3

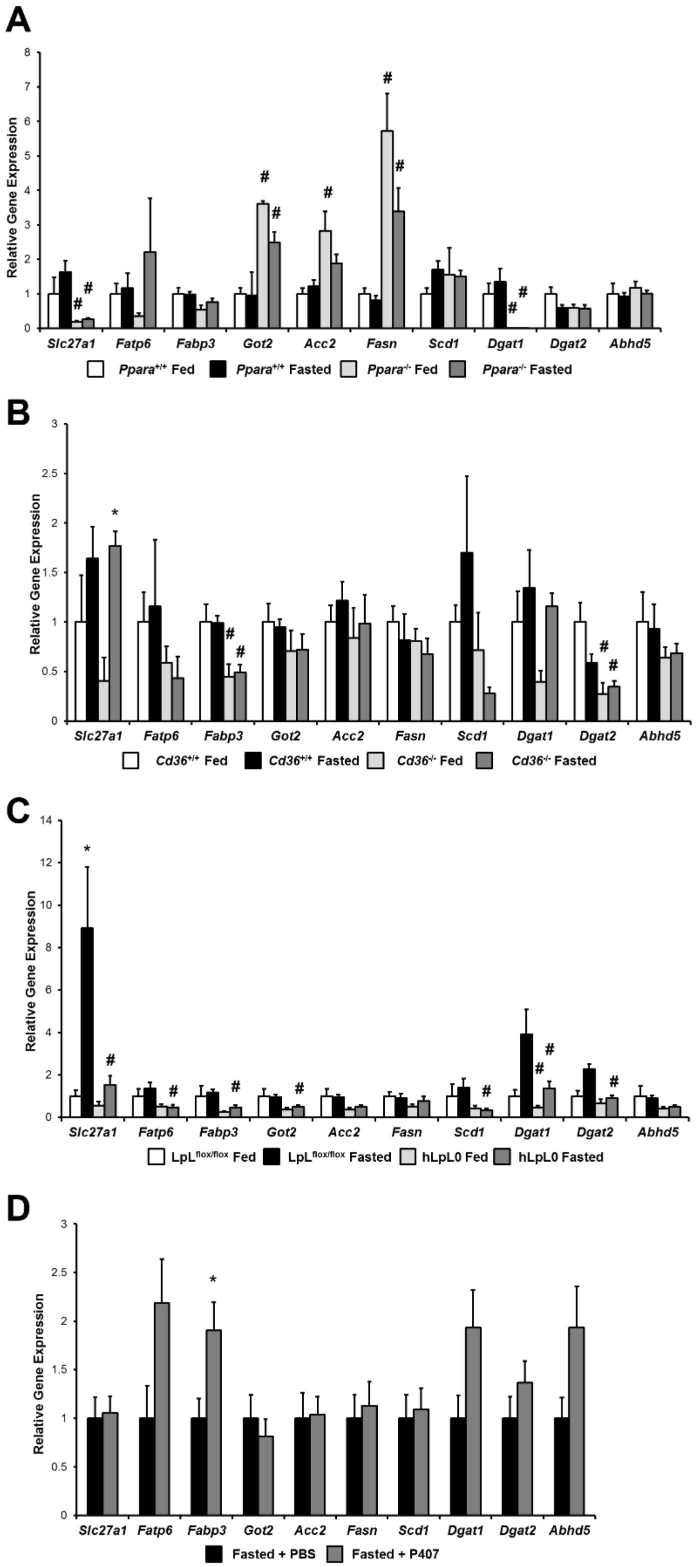

Supplemental Figure 4

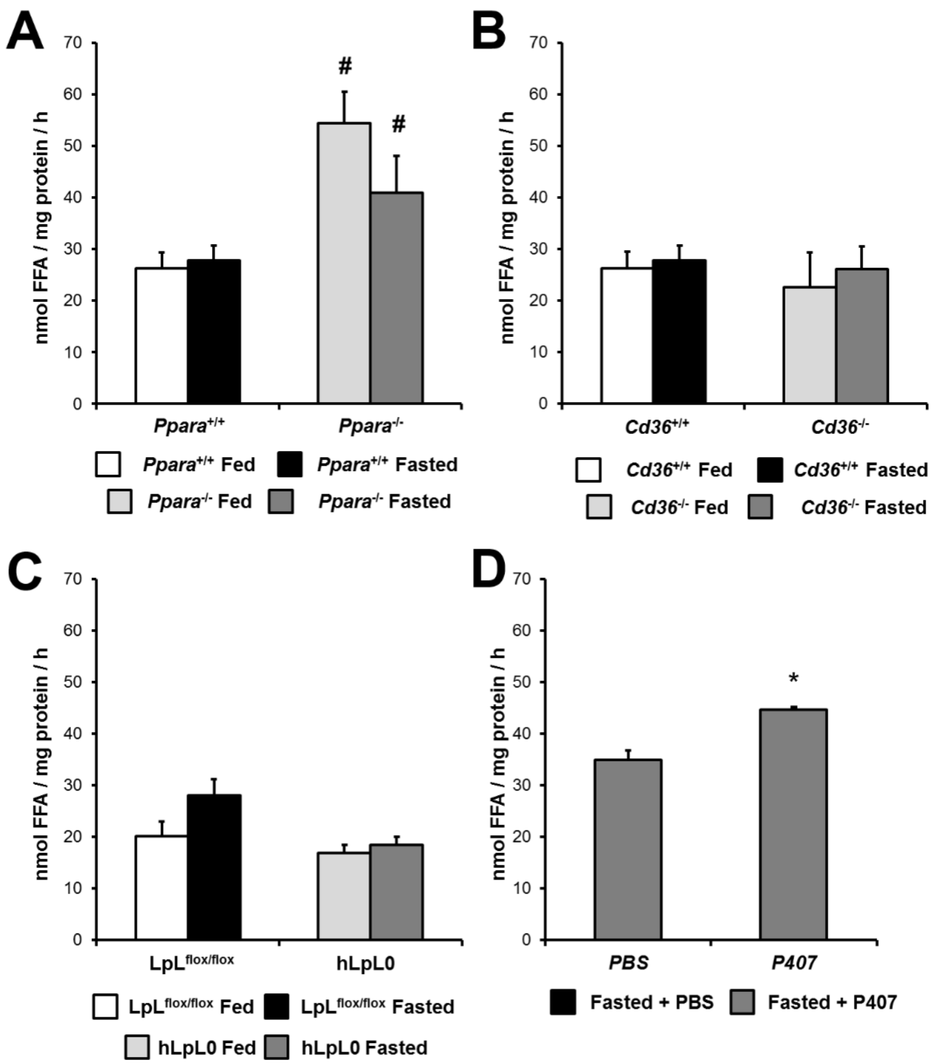

Supplemental Table 1. Mouse primers used for qRT-PCR gene expression analysis

Gene	Forward primer	Reverse primer
18s	5'-CCATCCAATCGGTAGTAGCG-3'	5'-GTAACCCGTTGAACCCCATT-3'
Abhd5	5'-TGACAGTGATGCGGAAGAAG-3'	5'-AGATCTGGTCGCTCAGGAAA-3'
Acc2	5'-TGGAGTCCATCTTCCTGTCC-3'	5'-GGACGCCATACAGACAACCT-3'
Acox1	5'-GGATGGTAGTCCGGAGAACA-3'	5'-AGTCTGGATCGTTCAGAATCAAG-3'
Atgl	5'-CGCCTTGCTGAGAATCACCAT-3'	5'-AGTGAGTGGCTGGTGAAAGGT-3'
Cd36	5'-TGTGTTTGGAGGCATTCTCA-3'	5'-TGGGTTTTGCACATCAAAGA-3'
Cpt1b	5'-TCTAGGCAATGCCGTTCAC-3'	5'-GAGCACATGGGCACCATAC-3'
Dgat1	5'-GTGCACAAGTGGTGCATCAG-3'	5'-CAGTGGGATCTGAGCCATCA-3'
Dgat2	5'-CTGTCACCTGGCTCAACAGA-3'	5'-TATCAGCCAGCAGTCTGTGC-3'
Fabp3	5'-GACGAGGTGACAGCAGATGA-3'	5'-TGCCATGAGTGAGAGTCAGG-3'
Fasn	5'-TTGCTGGCACTACAGAATGC-3'	5'-AACAGCCTCAGAGCGACAAT-3'
Fatp6	5'-GGTCACGGTGCTGGATAAGT-3'	5'-CGAGGAGTGGTTCAGGAGAG-3'
Glut1	5'-GCTGTGCTTATGGGCTTCTC-3'	5'-CACATACATGGGCACAAAGC-3'
Glut4	5'-ACTCTTGCCACACAGGCTCT-3'	5'-CCTTGCCCTGTCAGGTATGT-3'
Got2	5'-GTTGAAATGGGACCTCCAGA-3'	5'-GGGCAGGTATTCTTTGTCCA-3'
Lpl	5'-GCTGGTGGGAAATGATGTG-3'	5'-TGGACGTTGTCTAGGGGGTA-3'
Pdk4	5'-TTCTCGGAGTCTGGAATGCT-3'	5'-GCTCTAGCCGAACACGAATC-3'
Plin2	5'-CTACGACGACACCGAT-3'	5'-CATTGCGGAATACGGAG-3'
Plin5	5'-GTGATCAGACAGCTCAGGACCCT-3'	5'-CGATTCACCACATTCTGCTGG-3'
Scd1	5'-TGCGATACACTCTGGTGCTC-3'	5'-TAGTCGAAGGGGAAGGTGTG-3'
Slc27a1	5'-TTCTCGGAGTCTGGAATGCT-3'	5'-GCTCTAGCCGAACACGAATC-3'

Supplemental Methods

Density Ultracentrifugation of Plasma Approximately 60 µL of mouse plasma was separated by density ultracentrifugation as previously described (1). Briefly, 60 µL of plasma was underlayed to 60 µL of 1.006 g/mL saline solution in an ultracentrifuge tube. Each tube was centrifuged at 70,000 RPM at 12°C for 3 hours. The supernatant was removed and contains VLDL. Next, 60 µL of 1.12 g/mL KBr solution was added to the remaining infranatant. This was centrifuged at 70,000 RPM at 12°C for 12 hours. The supernatant was removed and contains LDL. The infranatant was removed and 60 µL of 1.34 g/mL mixture of KBr and NaCl added. This was centrifuged at 70,000 RPM at 12°C

for 12 hours. The supernatant was removed and contains HDL. The remaining infranatant contains all other plasma proteins, including FFA complexed to albumin. Counts of [^{3}H]-oleate was measured in each fraction and reported as percentage of total counts measured.

Ceramide Quantification

Ceramides were measured as previously described (2). All solvents for sample extraction and LC/MS were LC/MS grade (or LC grade when LC/MS grade was not available) and were purchased from Fisher Scientific (Pittsburgh, PA, USA). Ceramide standards were purchased from Avanti Polar Lipid, Inc. (Alabaster, AL, USA). Samples were processed as described previously with modification (2). Briefly, 3 ml of the methanol containing 20 µl of a 2 µM internal standard mixture (Avanti LM-6002, containing C12 and C25 ceramides) were added to 100 µl aqueous of heart homogenate containing 10 mg heart tissue in a clean glass tube, vortex-mixed and allowed equilibrate for 10 minutes on ice. Then the mixture was vortexed again and centrifuged at 3,000g for 10 minutes at 4 C degree. The organic upper phase was transferred to a second clean glass tube and evaporated under nitrogen. The extracted lipids were reconstituted in 300 µl of methanol: acetonitrile (v:v=1:1) and transferred to LC/MS autosampler vials (Waters, P/N 600000670CV) for evaporation under nitrogen. The lipid extract was finally reconstituted in 50 µl methanol for injection. All LC/MS/MS running were carried out on a Waters Xevo TQ MS ACQUITY UPLC system (Waters, Milford, MA) controlled by Mass Lynx Software version 4. 1. The sample was maintained at 4°C in the autosampler and 5 µl was loaded onto a Waters ACQUITY UPLC BEH Phenyl column (3 mm inner diameter × 100 mm with 1.7 µm particles), preceded by a 2.1 × 5 mm guard column containing the same packing.

The UPLC flow rate was continuously 300 µl/min in a binary gradient mode with methanol and water both containing 0.2% formic acid and 1 mM ammonium formate. Positive ESI-MS/MS mass spectrometry was performed to identify ceramide species of interest. Different species were confirmed by comparing the retention times of experimental compounds with those of authentic standards. Concentrations of ceramides in the samples were quantified by comparing integrated peak areas for those of each ceramide against those of known amounts of purified standards. Loss during extraction was accounted for by adjusting for the recovery of the internal standard added before extraction.

Triglyceride lipase activity assay

TG lipase activity was performed as described (3, 4). Tissue lysates were prepared in solution A (0.25 M sucrose, 1 mM EDTA, 1 mM DTT, pH 7.0, 1 µg/mL pepstatin, 2 µg/mL antipain, and 20 µg/mL leupeptin). Protein concentration was measured with Bradford reagent, and 50 µg of protein was used for each assay. Micelles were prepared by sonication in 0.1 M potassium phosphate buffer (pH 7.0) with 1.67 mM triolein, 10 µCi [9,10-^{3}H(N)] triolein/mL (Perkin-Elmer), 142.5 mM phosphatidylcholine, and 47.5 mM phosphatidylinositol. Fatty acid free BSA was added to the micelles at 2% concentration (weight/volume). 100 µL of radiolabeled substrate was incubated with diluted protein lysates for 1 hour at 37°C in a 125 RPM shaking incubator. The reaction was stopped by the addition of 3.25 mL mixture of methanol, chloroform, and n-heptanes (10:9:7, volume). Fatty acids were extracted by addition of 1 mL of 0.1 M potassium carbonate, 0.1 M boric acid (pH 10.5) and centrifugation at 3000 RPM for 10 minutes. 200 µL of the aqueous phase were counted for radioactivity.

Supplemental References

1. Iqbal, J., K. Anwar, and M. M. Hussain. 2003. Multiple, independently regulated pathways of cholesterol transport across the intestinal epithelial cells. *J Biol Chem* **278**: 31610-31620.

2. Clugston, R. D., H. Jiang, M. X. Lee, R. Piantedosi, J. J. Yuen, R. Ramakrishnan, M. J. Lewis, M. E. Gottesman, L. S. Huang, I. J. Goldberg, P. D. Berk, and W. S. Blaner. 2011. Altered hepatic lipid metabolism in C57BL/6 mice fed alcohol: a targeted lipidomic and gene expression study. *J Lipid Res* **52**: 2021-2031.

3. Pollak, N. M., M. Schweiger, D. Jaeger, D. Kolb, M. Kumari, R. Schreiber, S. Kolleritsch, P. Markolin, G. F. Grabner, C. Heier, K. A. Zierler, T. Rulicke, R. Zimmermann, A. Lass, R. Zechner, and G. Haemmerle. 2013. Cardiac-specific overexpression of perilipin 5 provokes severe cardiac steatosis via the formation of a lipolytic barrier. *J Lipid Res* **54**: 1092-1102.

4. Zierler, K. A., D. Jaeger, N. M. Pollak, S. Eder, G. N. Rechberger, F. P. Radner, G. Woelkart, D. Kolb, A. Schmidt, M. Kumari, K. Preiss-Landl, B. Pieske, B. Mayer, R. Zimmermann, A. Lass, R. Zechner, and G. Haemmerle. 2013. Functional cardiac lipolysis in mice critically depends on comparative gene identification-58. *J Biol Chem* **288**: 9892-9904.
